# Epilepsy in Tanzanian children: Association with perinatal events and other risk factors

**DOI:** 10.1111/j.1528-1167.2011.03395.x

**Published:** 2012-02-06

**Authors:** Kathryn J Burton, Jane Rogathe, Roger Whittaker, Kshitij Mankad, Ewan Hunter, Matthew J Burton, Jim Todd, Brian G R Neville, Richard Walker, Charles R J C Newton

**Affiliations:** * Kilimanjaro Christian Medical CentreMoshi, Tanzania; † Neurosciences Unit, Institute of Child Health, University College LondonLondon, United Kingdom; ‡ Northumbria Healthcare NHS Foundation Trust, North Tyneside General HospitalNorth Shields, United Kingdom; § Great Ormond Street HospitalLondon, United Kingdom; ¶ Department of Clinical Research, Faculty of Infectious and Tropical Diseases, London School of Tropical MedicineLondon, United Kingdom; # National Institute for Medical ResearchMwanza, Tanzania; ** Department of Paediatrics, Muhimbili University of Health and Allied SciencesDar-es-Salaam, Tanzania; †† Department of Psychiatry, University of OxfordOxford, United Kingdom

**Keywords:** Epilepsy, Africa, Children, Etiology, Adverse perinatal events, Prevalence

## Abstract

**Purpose:**

To define the prevalence and risk factors for epilepsy in children in a rural district of Tanzania by conducting a community-based case–control study.

**Methods:**

Children aged 6–14 years with active epilepsy (at least two unprovoked seizures in the last 5 years) were identified in a cross-sectional survey in Tanzania. Cases were compared with age-matched controls.

**Key Findings:**

Overall 112 children with epilepsy (CWE) were identified; the unadjusted prevalence of epilepsy was 2.91 per 1,000 (95% confidence interval [95% CI] 2.4–3.5). The main seizure types were focal motor with secondary generalization in 73 (65.2%) of 112 and generalized convulsive seizures in 19 (16.9%) of 112. Adverse perinatal events were present in 16 (14%) of 112 cases but in no controls. In multivariate analysis, epilepsy was associated with number of parents who were resident at home (odds ratio [OR] 6.2 for none vs. both resident, 95% CI 1.5–25.5), history of adverse perinatal events (OR 14.9, 95% CI 1.4–151.3), family history of afebrile seizures (OR 5.7, 95% CI 1.0–27.5), and poor scholastic attainment (OR 8.6, 95% CI 4.0–18.4). Electroencephalography (EEG) and computed tomography (CT) scans were abnormal in 44 (44%) of 101 and 26 (29%) of 90 cases, respectively. Overall, 98 (88%) of 112 cases had focal features on assessment.

**Significance:**

In this study from sub-Saharan Africa, CWE predominantly had focal features that support the suggestion that most epilepsy in this region has a symptomatic etiology. Adverse perinatal events were strongly associated with epilepsy. Genetic and social factors may also be important. Epilepsy may be preventable in a significant proportion of children with better antenatal and perinatal care.

Epilepsy is one of the most common neurological disorders worldwide, and the prevalence of active epilepsy is significantly higher in developing than in developed countries, especially in rural areas (Ngugi et al., 2010). A review of the epidemiology and etiology of epilepsy in sub-Saharan Africa (SSA) in 2005 documented that there were many descriptive studies of etiology of epilepsy, but only a few case–control studies (Preux & Druet-Cabanac, 2005). Most did not provide electroencephalography (EEG) or neuroimaging data. Since then, there have been further descriptive studies from Africa of risk factors associated with epilepsy (Prischich et al., 2008; Simms et al., 2008; Ogunlesi et al., 2009; Winkler et al., 2009; Duggan, 2010) but only two population-based case–control studies—both from Kenya (Edwards et al., 2008; Mung’ala-Odera et al., 2008). Studies from other parts of SSA are required to identify the range of preventable causes of childhood epilepsy in the region. Therefore, we performed a case–control study in Tanzania to identify risk factors for childhood epilepsy and to examine EEG and neuroimaging findings in children with epilepsy.

## Methods

### Study area and population

We conducted a cross-sectional study of epilepsy in an established demographic surveillance site (DSS) in Hai district, Northern Tanzania (AMMP, 1997). We identified all 6–14-year-old children with epilepsy after a census in January 2009. We used age-matched controls selected from Hai for comparison.

### Definitions

We used the International League Against Epilepsy (ILAE, 1993) definitions and defined active epilepsy as two or more afebrile seizures, at least 24 h apart, unrelated to acute infection, metabolic disturbance, neurologic disorders or drugs, in the last 5 years. Children who were on antiepileptic drugs were also considered as having active epilepsy. Epileptic seizures were classified according to the ILAE guidelines (ILAE, 1997). Seizure etiology was categorized as idiopathic or structural if there was sufficient evidence from history and examination to assess for an underlying cause for epilepsy and as undetermined if there were insufficient data (Thurman et al., 2011). Birth histories including a combination of prolonged labor, delayed crying, and encephalopathy with poor feeding were categorized as being suggestive of hypoxic-ischemic encephalopathy (HIE).

### Case ascertainment and criteria for inclusion and exclusion

During the January 2009 census, a nine-item, previously validated questionnaire to detect epilepsy with partial and generalized seizures was administered to all households in Hai district (Placencia et al., 1992). This instrument was translated into Swahili and then back translated. The study pediatrician (KJB), who has training in pediatric epilepsy, assessed each child who responded positively to the questionnaire or those brought by village enumerators who acted as key informants. Enumerators had been trained in the presentation of epilepsy and were asked to bring forward any children in the community who they felt might have epilepsy. The prevalence date was June 2009 and the confirmation of case and control status and the assessment of risk factors was performed in the community between June and December 2009.

The diagnosis of active epilepsy, likely etiology, and seizure type were verified by a pediatric neurologist (CN) and by a second independent pediatric neurologist (BN) for cases that were considered indeterminate. Where more than one seizure type occurred, the most frequent was coded. For the purpose of this study, cases of epilepsy were defined as children with active epilepsy aged 6–14 years who were resident in Hai at the time of the census. Those children for whom consent was refused or who were younger than 6 years old were excluded. We limited inclusion to children aged 6–14 years of age to reliably exclude those with febrile seizures in younger children and to focus on risk factors for childhood epilepsy.

### Controls

Controls were drawn from a random sample selected from all the children aged 6–14 years who were resident in Hai at the census. Controls were identified through the census by matching for age (±1 year), sex, and village to the positive responders. From this list of eligible children, we estimated that 186 controls were required to account for the likely 25% refusal rate, to give at least one control for each case.

### Neuropediatric assessment

For each case and control, a full clinical history and neuropediatric examination was completed by the same pediatrician (KB) using a standardized questionnaire. EEG and computerized tomography (CT) scan were offered to every case at recall. EEG was performed using a Nihon Kohden (Tokyo, Japan) Neurofax 11000K machine. Twenty leads were attached using a standard montage. Patients had EEG recorded while asleep and awake, with hyperventilation and photic stimulation. A neurophysiologist (RW) in the United Kingdom reported the EEG results using a standardized form. The CT scans were performed with contrast on a Philips (Andover, MA, U.S.A.) Tomoscan 4000 machine. CT scans were reported locally to exclude any acute pathology. They were then reported using a standardized format by a pediatric neuroradiologist (KM) in the United Kingdom.

### Cognitive assessment

An assessment of cognitive function was made using the Goodenough-Harris Drawing Test (GHDT) (Harris, 1963). The GHDT was used because it has good reliability and validity compared to other cognitive tests including the Wechsler Intelligence Scale for Children-Revised (WISC) and the Stanford-Binet Intelligence Scale (Abell et al., 1996). GHDT scores had a high interrater reliability coefficient (0.96) and correlated significantly with Full Scale intellectual quotient (IQ) on the WISC (r = 0.64, p < 0.05) (Gayton et al., 1974). Each drawing was marked by one person (KB). Raw scores were age-standardized and used as an estimate of IQ.

### Ethical approval

Approval for this study was obtained from the National Institute for Medical Research (NIMR) in Tanzania and locally from the Ethics Committee of Kilimanjaro Christian Medical Centre (KCMC), Moshi. Parents and guardians of cases and controls were given written and verbal information in Kiswahili before signing consent forms on which they agreed to participate.

### Data analysis

All data was double-entered into a Microsoft Access database (2007 version; Microsoft Corporation, Redmond, WA, U.S.A.). The two database copies were compared using Epidata (Version 3.1; Epidata Association, Odense, Denmark) and each discrepancy was checked against the original data forms. Statistical analysis was performed using STATA v.10 (StataCorp, College Station, TX, U.S.A.).

Ethnic groups were classified as Chagga as the predominant ethnic group compared to all others. Education of the head of household was used as a surrogate marker for socioeconomic status, as previous research from Hai had shown that this was a key determinant in explaining the between-household variation in expenditure (Adult Morbidity and Mortality Project Team, 2003). Children whose mother or father was single, separated, divorced, or widowed were designated as having only one parent resident at home; those who were orphans or had been left with distant relatives were classified as having no parents resident at home. Children with a combination of history of prolonged labor, delayed crying, poor feeding, or being admitted to the neonatal unit were designated as having had an adverse perinatal event. If one or more parent was absent from home, then history of perinatal events was obtained from the other parent or close relative. Those with insufficient reliable data from history (seven controls and seven cases) were excluded from the univariate and multivariate analyses. Scholastic attainment was used as an indicator of functional outcome of a child’s ability and was considered poor if the child was not in an age-appropriate class Multivariable logistic regression models were developed using p-values of ≤0.2 in the univariate analyses. Adverse perinatal events occurred only in cases, so odds ratios (ORs) were calculated 10 times (switching one different control each time to adverse perinatal event present) and the average OR determined. The range of 95% confidence intervals (95% CIs) and the least significant p-value were quoted after these adjustments. For comparing those with and without cerebral palsy, one control was allocated to have an adverse perinatal event and cerebral palsy.

We expected at least 100 CWE, which with a one-to-one matching would have 80% power to detect a significant association between a risk factor with a prevalence of 10% in controls with an OR of 2.5.

## Results

### Study subjects

**Figure 1:**
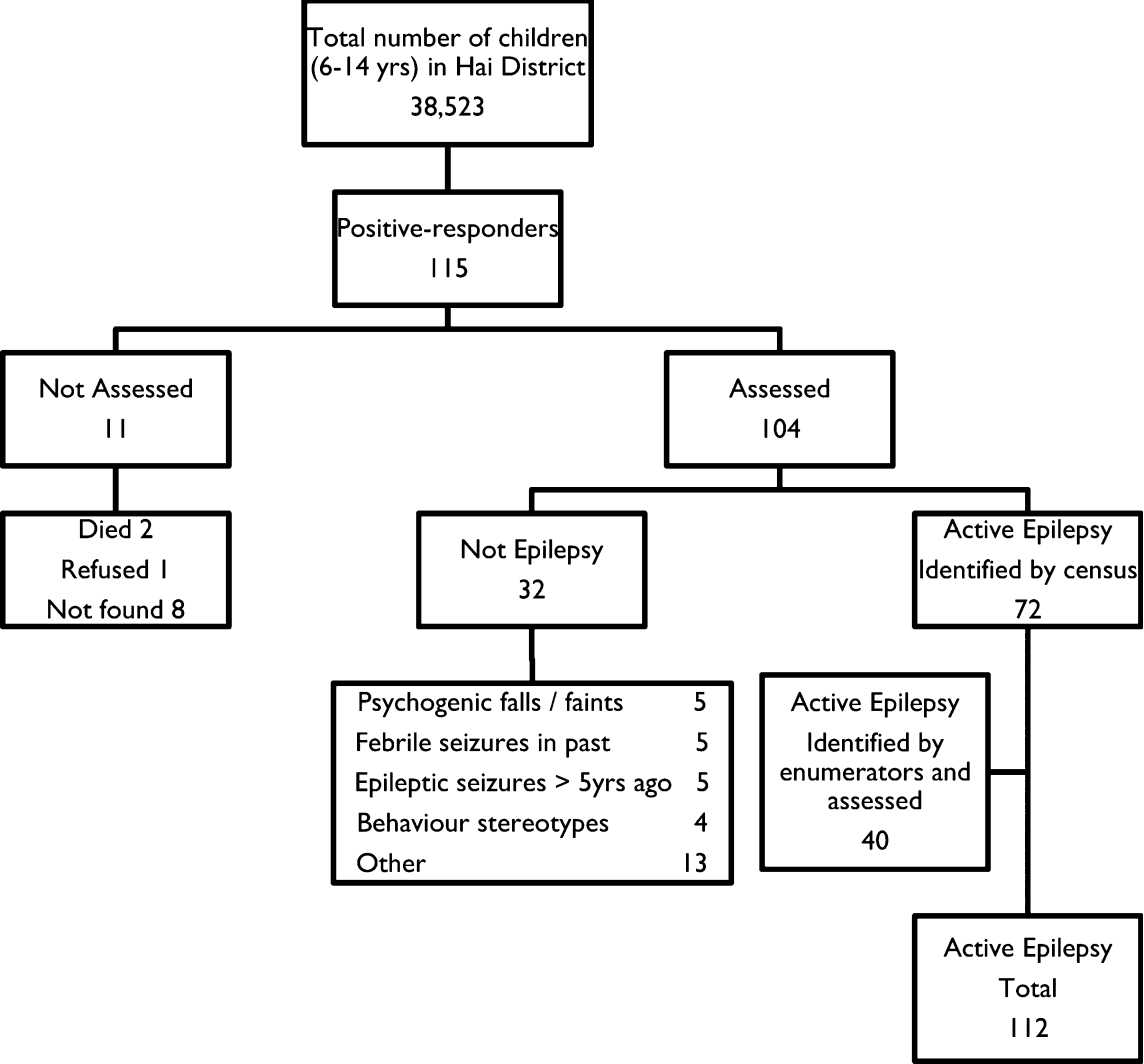
Flow chart of case ascertainment and recruitment.

Overall 112 children with active epilepsy and 113 controls were identified (Fig. 1). For one case only (girl, aged 10 years) care providers refused consent. Demographic characteristics are presented in Table 1; age, sex, consanguinity, and head of the household education were comparable between cases and controls. The proportion of cases coming from the Chagga ethnic group was lower than that for controls (OR 2.5, 95% CI 1.2–5.3, p = 0.014). From the list of 186 matched controls, 73 (39.2%) children were not found or their care provider refused consent. There was no significant difference between the controls who were seen and the controls who were not (median age 11 years for both; and 52.2% and 46.6% were male, respectively; OR 1.3, 95% CI 0.7–2.3, p = 0.453).

**Table 1:** Demographic characteristics of cases and controls

Variable	Cases (n = 112) n (%)	Controls (n = 113) n (%)
Sex, male	57 (50.9)	57 (50.4)
Median age (years, interquartile range)	12 (10, 13)	12 (9, 13)
Education of head of house		
None	6 (5.4)	3 (2.7)
Primary	93 (83.0)	90 (79.6)
Secondary	11 (9.8)	12 (10.6)
Not known	2 (1.8)	8 (7.1)
Ethnic group		
Chagga	86 (76.8)	101 (89.4)
Other	26 (23.2)	12 (10.6)
Consanguinuity		
None	102 (91.1)	101 (89.4)
Present	2 (1.8)	0
Not known	8 (7.1)	12 (10.6)

### Prevalence of epilepsy

There were 38,523 children aged 6–14 years (23.9% of total population of 161,119) identified as resident in the Hai census during 2009, of whom 50.6% were male. Of these children, 112 were found to have active epilepsy, with an unadjusted prevalence of 2.91 (95% CI 2.4–3.5) per 1,000 for active epilepsy.

### Classification of seizures and age at onset

In the 112 children with epilepsy, the seizure types identified were focal motor with secondary generalization in 73 (65.2%), generalized convulsive seizures (tonic–clonic and clonic) in 19 (16.9%), complex partial in 11 (9.8%), myoclonic in 2 (1.8%), focal motor in 3 (2.7%), generalized absence in 1 (0.9%), and undetermined in 3 (2.7%). Onset of seizures before 3 years of age was found in 40 (35.7%) 112.

In cases, the probable etiology from the clinical history was idiopathic in 56 (50.0%), hypoxic-ischemic encephalopathy (HIE) in 10 (8.9%), intracranial infection in 9 (8.0%), head injury 3 (2.7%), neurocutaneous disorder (tuberous sclerosis, neurofibromatosis, and Sturge-Weber) in 3 (2.7%), other structural causes in 19 (17.0%), and undetermined in 12 (10.7%).

### Risk factors associated with epilepsy

The prevalence of possible risk factors for epilepsy in cases and controls are shown in Table 2. Among cases, 16 (14.3%) of 112 children had a history of adverse perinatal events and none of the controls did. Of these events, 6 had HIE, all of whom developed a motor disorder (four with hemiplegia and two with diplegia) and all had an IQ <60. Three additional patients with other perinatal events developed cerebral palsy and all with cognitive impairment. Only 3 of the 16 patients had IQs >90 (Table 3). The univariate and multivariable associations are shown in Table 4. In the multivariable model, epilepsy was strongly associated with history of adverse perinatal event, family history of nonfebrile seizures, poor scholastic attainment, and with one or no parent resident at home. Epilepsy was strongly associated with the presence of cerebral palsy on univariate analysis (OR 22.9, 95% CI 3.0–174.1, p = 0.003). After including cerebral palsy in the multivariable model, adverse perinatal events were still significantly associated with epilepsy (OR 10.2, 95% CI 1.1–93.4, p = 0.040).

**Table 2:** Characteristics of children with epilepsy and controls

Characteristics	Cases (n = 112) n (%)	Controls (n = 113) n (%)
Sex		
Male	57 (50.9)	57 (50.4)
Female	55 (49.1)	56 (49.6)
Age at assessment (years)		
<12 years	73 (65.2)	75 (66.4)
12 years and older	39 (34.8)	38 (33.6)
Ethnic group (Chagga)		
Chagga	86 (76.8)	101 (89.4)
Other	26 (23.2)	12 (10.6)
Religion (Christian)		
Christian	91 (81.3)	90 (79.7)
Muslim and other	21 (18.7)	23 (20.3)
Parents resident at home		
Both	71 (63.4)	89 (78.8)
One parent	26 (23.2)	18 (15.9)
None	14 (12.5)	5 (4.4)
Not known	1 (0.9)	1 (0.9)
Education of head of house		
None	6 (5.4)	3 (2.7)
Primary	93 (83.0)	90 (79.6)
Secondary	11 (9.8)	12 (10.6)
Not known	2 (1.8)	8 (7.1)
Education of mother		
None	8 (7.1)	3 (2.7)
Primary	82 (73.3)	95 (84.1)
Secondary	8 (7.1)	5 (4.4)
Not known	14 (12.5)	10 (8.8)
Adverse perinatal event		
None	89 (79.5)	106 (93.8)
Occurred	16 (14.3)	0 (0.0)
Not known	7 (6.2)	7 (6.2)
Head injury		
None	105 (93.7)	111 (98.2)
Occurred	4 (3.6)	1 (0.9)
Not known	3 (2.7)	1 (0.9)
Family history of nonfebrile seizures in first-degree relative		
None	96 (85.7)	109 (96.4)
Present	13 (11.6)	3 (2.7)
Not known	3 (2.7)	1 (0.9)
Past history of febrile seizures		
None	88 (78.6)	99 (87.6)
Present	16 (14.3)	8 (7.1)
Not known	8 (7.10)	6 (5.3)
Poor scholastic attainment		
None	41 (36.6)	92 (81.4)
Present	71 (63.4)	21 (18.6)
Past history of malaria with seizures and/or coma		
Never	103 (91.9)	105 (93.7)
Occurred	7 (6.3)	7 (6.3)
Not known	2 (1.8)	0 (0.0)

**Table 3:** Details of 16 cases with adverse perinatal events

History of adverse event	Seizure type	Motor problems	CT scan result	EEG	Estimated IQ on GHDT
One of triplets (2 others died), born at 32 weeks	Generalized clonic	None	Normal	Asymmetrical focal features with multifocal spikes in frontal region	<50
History strongly suggestive of HIE	Focal onset	Right hemiplegia	Not done	Uninterpretable	<50
Mother bled in pregnancy, born at 28 weeks	Focal onset	Spastic quadriplegia	Generalized lack of white matter	Multifocal spikes in posterior temporal and occipital regions	<50
Mother had malaria so born 1.9 kg at 36 weeks	Focal onset	None	Normal	Normal	90
History suggestive of HIE	Focal onset	Spastic diplegia	Normal	Multifocal epileptiform abnormalities	53
Born at 32 weeks, admitted and had difficulty breathing	Focal onset	None	Normal	Normal	96
Born at 36 weeks; had neonatal fever and poor feeding	Focal onset	Spastic diplegia	Normal	Multifocal spikes, right central focus	<50
Admitted with fever and severe jaundice on second day after birth	Focal onset	Choreoathetoid cerebral palsy	Right frontal lobe atrophy	Abnormal EEG with moderate encephalopathy	<50
History suggestive of HIE	Focal onset	Right hemiplegia	Normal	Normal	57
History suggestive of HIE	Focal onset	Right hemiplegia	Not done	None done	50
History suggestive of HIE	Focal onset	Spastic diplegia	Normal	Asymmetrical extratemporal focal spikes	<50
Blue at birth, took 1 h to cry after birth	Focal onset	None	Normal	Normal	59
Second born of twins, abruption at delivery	Focal onset	None	Normal	Normal	58
Neonatal sepsis	Focal onset	None	Not done	Not done	On clinical assessment, had moderate cognitive impairment with expressive language difficulty
Caesarean section for prolonged rupture of membranes and subsequently fed poorly	Generalized clonic	None	Not done	Normal	101
History of HIE	Focal onset	Right hemiplegia	Left posterior cerebral artery territory infarct	Asymmetrical focal temporal spikes, left centrotemporal focus	<50

HIE, hypoxic-ischemic encephalopathy; GHDT, Goodenough Harris drawing test.

**Table 4:** Univariate and multivariate analyses of risk factors for the development of epilepsy

Variable	OR	95% CI	p-Value
Univariate associations with epilepsy			
Sex (male)	1.0	0.6–1.7	0.946
Age at assessment (12 years and older)	1.1	0.6–1.8	0.850
Ethnic group (not Chagga)	2.4	1.2–5.3	0.014
Religion (Christian)	1.1	0.6–2.1	0.762
Parents resident at home (both parents)	1.0	–	–
Parents resident at home (one parent)	1.8	0.9–3.6	0.086
Parents resident at home (none)	3.5	1.2–10.2	0.021
Education of head of house (none)	1.0	–	–
Education of head of house (primary)	0.5	0.1–2.1	0.361
Education of head of house (secondary)	0.5	0.1–2.3	0.342
Education of mother (none)	1.0	–	–
Education of mother (primary)	0.5	0.2–1.7	0.295
Education of mother (secondary)	1.7	0.3–9.4	0.564
Adverse perinatal event	18.9	2.4–146.5	0.005
Head injury (occurred)	4.2	0.5–38.4	0.200
Family history of nonfebrile seizures (present)	4.9	1.4–17.8	0.015
Past history of febrile seizures (present)	2.25	0.91–5.5	0.076
Poor scholastic attainment (present)	7.6	4.1–14.0	<0.001
Malaria with seizures and/or coma (occurred)	1.0	0.3–3.0	0.972
Multivariable logistic regression model for epilepsy			
Ethnic group (not Chagga)	2.4	0.9–6.4	0.084
Parents resident at home (both parents)	1.0	–	–
Parents resident at home (one parent)	2.8	1.1–6.5	0.023
Parents resident at home (none)	6.2	1.5–25.5	0.011
Adverse perinatal event	14.9	1.4–151.3	0.024
Head injury (occurred)	7.6	0.6–97.3	0.119
Family history of nonfebrile seizures (present)	5.7	1.02–27.5	0.046
History of febrile seizures	2.4	0.8–7.0	0.117
Poor scholastic attainment (present)	8.6	3.9–18.4	<0.001

### Neurophysiology and neuroradiology

**Figure 2:**
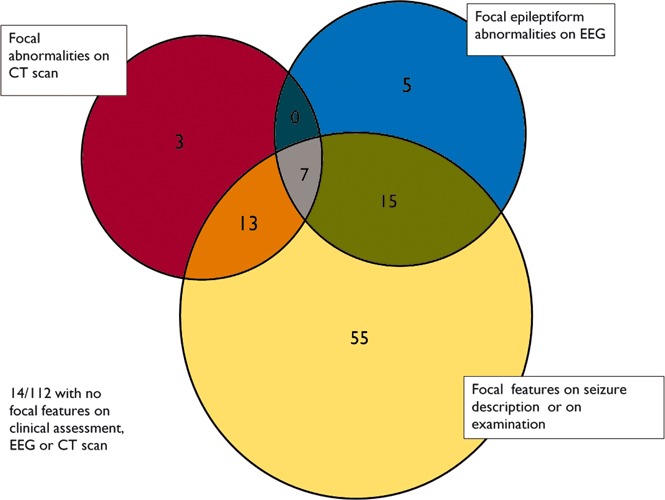
Diagram of focal features on clinical history and examination compared to focal abnormalities on EEG and CT scan.

Of 112 cases, 101 had EEG performed (10 missed recall, one EEG not tolerated). EEG abnormalities were seen in 44 (43.6%) of 101 and were focal in 27 (61.4%) of 44. Among cases, 90 of 112 had CT scans of which 26 (28.9%) of 90 were abnormal. The abnormal EEG and CT scan results are shown in Tables 5 and 6. Those who did and did not have imaging were similar, except in those with imaging, the head of households had completed more education (p = 0.029). Overall, 98 of (87.5%) 112 cases had focal features on at least one of clinical assessment, CT or EEG (Fig. 2).

**Table 5:** Abnormal EEG results

Type of EEG abnormalities	n (%)
Generalized epileptiform abnormalities	9 (20.5)
Multifocal epileptiform abnormalities	11 (25.0)
Temporal lobe abnormalities	7 (15.9)
Extratemporal focal abnormalities	9 (20.5)
Generalized nonepileptiform abnormalities	8 (18.1)
Total	44 (100)

**Table 6:** Abnormal CT scan results

Type of abnormality on CT scan	n (%)
Focal cerebral atrophy	5 (19.3)
Cerebellar/brainstem atrophy	4 (15.4)
Porencephalic cyst	2 (7.7)
Generalized lack of white matter bulk	3 (11.5)
Calcified lesion (undetermined)	2 (7.7)
Neurocysticercosis	2 (7.7)
Pre/perinatal vascular event	5 (19.3)
Previous tuberculous meningitis	1 (3.8)
Sturge-Weber	1 (3.8)
Tuberous sclerosis	1 (3.8)
Total	26 (100)

## Discussion

This community-based study of CWE in SSA found that most epilepsy was of focal onset and that development of epilepsy was strongly associated with adverse perinatal events.

### Seizure type

The most common seizure type was partial motor seizures with secondary generalization. Overall three-fourths of the cases had partial seizures with or without secondary generalization, and overall 88% had focal features. This finding concurs with results of other studies from sub-Saharan Africa in which at least one third are clinically classified as partial seizures becoming generalized (Dent et al., 2005; Edwards et al., 2008; Mung’ala-Odera et al., 2008). The proportion of seizures that are partial in onset can be underestimated in studies, as clinical history and investigations may be limited. A more recent study of 6–18-year-old children from rural Kenya, found that 71% with active convulsive epilepsy had evidence of focal neurologic abnormality documented by seizure description, focal neurologic deficits, or by focal abnormalities on the EEG (Munyoki et al., 2010).

### Risk factors for epilepsy in children in sub-Saharan Africa

Our study found a very strong association between epilepsy and adverse perinatal events, even though this was based upon maternal recall only. This association had wide confidence intervals, and there were no adverse perinatal events in controls, which may be a reflection of the small sample size. In a community-based study in America, adverse perinatal events were a risk factor for cerebral palsy but not for epilepsy only (Nelson & Ellenberg, 1984). However, the association with adverse perinatal events was still significant after controlling for the presence of cerebral palsy, suggesting an independent effect in our population. This association is also consistent with the findings of other community-based studies from high-income countries (HICs) (Whitehead et al., 2006) and in particular SSA, such as Kenya (OR 5.7, 95% CI 2.6–12.7) (Edwards et al., 2008), Burundi (Nsengiyumva et al., 2003), and Tanzania (OR 7.3, 95% CI 2.2–25.2) (Matuja et al., 2001). Our study confirms this strong association in this region.

The severity of the brain insults seems to be similar to those reported in HICs, with a high prevalence of HIE and very high prevalence of cognitive impairment. In HICs, studies have shown an increased risk of developing epilepsy with both HIE (Bergamasco et al., 1984) and periventricular leukomalacia (Humphreys et al., 2007). Nine patients with adverse perinatal events had cerebral palsy, although the type of motor disorder did not necessarily conform to that expected from the insult (Stanley et al., 2000). Generally, cerebral palsy caused by HIE would be expected to be of the spastic quadriplegic or dyskinetic type (Hankins & Speer, 2003). It may be that some of the cases with a history suggestive of HIE had another condition such as infection that had predisposed them to hypoxia at birth. The association with adverse perinatal events would need to be examined in a larger population of CWE to confirm its validity at a population level in SSA. Adverse perinatal events are common in SSA, where there is limited obstetric care, and are known to be potentially avoidable if antenatal services can be improved (Islam & Yoshida, 2009).

Family history of nonfebrile seizures had a strong association with epilepsy (OR 5.7, 95% CI 1.02–27.5). The association with a positive family history is a consistent finding in other studies from Kenya (Edwards et al., 2008), Tanzania (Matuja et al., 2001), Burundi (Nsengiyumva et al., 2003), and Ethiopia (Tekle-Haimanot et al., 1997). This implies that genetic factors have a significant role in etiology, although common environmental risk factors cannot be excluded.

In our study, children who had one (mother or father) or no parent resident at home were more likely to have epilepsy. This adds to the findings of a community-based study of risk factors for epilepsy in rural Kenya in which epilepsy in children was associated with the child’s mother being a widow (Edwards et al., 2008). The association with absent parents may be consequential, as parents may abandon disabled children or may have died from other conditions including epilepsy. It may also be related to HIV infection, but we were unable to prove this without permission to test for HIV.

Epilepsy was also associated with poor scholastic attainment, which is a marker for learning difficulties. This has been found in other studies from the region in adults and in children (Matuja et al., 2001; Munyoki et al., 2010). It is likely that this relationship is not causal but is due to the same etiology for both epilepsy and cognitive impairment. Evidence for this is shown in studies that found that cognitive impairment predated the onset of seizures (Oostrom et al., 2003; Hermann et al., 2006).

Falciparum malaria is known to be a risk factor for developing subsequent epilepsy (Carter et al., 2004; Ngoungou & Preux, 2008). In our study, no significant association with malaria was found. However, 8% of patients (cases) gave a history of a severe febrile illness shortly before the onset of epilepsy. It was impossible to retrospectively determine the cause of fever. Therefore, it is likely that we have underestimated the effect of falciparum malaria.

The relationship between epilepsy and previous febrile seizures as a young child is complex. Studies from sub-Saharan Africa have shown conflicting findings (Ogunniyi et al., 1987; Matuja et al., 2001; Mung’ala-Odera et al., 2008). This may be due to differences in study populations and recall bias. Retrospective recall of febrile seizures has been shown to be unreliable (Sillanpaa et al., 2008). Variable associations have been found with prior head injury and epilepsy in the region (Ogunniyi et al., 1987; Nsengiyumva et al., 2003; Edwards et al., 2008; Munyoki et al., 2010). Our study does show some nonsignificant association between epilepsy and both head injury and febrile seizures; for a larger study with more power, these associations may have been significant.

### Neurophysiology and neuroimaging

Our community-based study of CWE found that nearly half of the EEG studies were abnormal, and of these, nearly two thirds had focal epileptiform abnormalities. This proportion is higher than in HICs (Shinnar et al., 1994), and there are few studies in CWE from Africa for comparison. A Nigerian study of 96 children referred to a tertiary hospital with recurrent seizures found 96% of this selected group had abnormal EEG studies and that focal abnormalities were very common (59%) (Osuntokun et al., 1974). A community-based rural study identified CWE who were aged 6–9 years old in Kenya, recorded EEGs on 80 children, and found abnormalities in 20% (Mung’ala-Odera et al., 2008).

In our study, nearly one third of cases had abnormal neuroimaging. This is greater than in community-based studies of CWE from HICs in which prevalence of structural abnormality ranges from 13–21% (Shinnar et al., 1999; Berg et al., 2000). The prevalence and type of neuroimaging abnormalities in children with epilepsy from Africa has not been studied. Children with neurologic impairment in rural Kenya had CT scans, and imaging abnormalities were found predominantly in those with motor deficits (82%) and were not found in those with seizures alone (Njuguna et al., 2007). The prevalence of neuroimaging abnormalities in our study is likely to be an underestimate, as CT is less sensitive than magnetic resonance imaging (Shinnar et al., 1999; Berg et al., 2000). We used CT in accordance with World Health Organization recommendations (World Health Organization, 2011), as CT is more available in Africa and is better at identifying neurocysticercosis, which is a known cause of epilepsy in this region (Quet et al., 2010).

The majority of CWE (88%) in our study had focal features in at least one of clinical assessment, EEG, or CT scan. A previous study from SSA also found a high prevalence of focal features in cases (Munyoki et al., 2010). These findings provide strong evidence that most of the epilepsy in the region is symptomatic and underscores the importance of identifying preventable causes.

### Prevalence of epilepsy in children in East Africa

The reported prevalence of active epilepsy in developing countries varies widely. The heterogeneity in prevalence can mostly be ascribed to study size (higher prevalence in smaller studies) and higher prevalence in rural areas (Ngugi et al., 2010). The unadjusted prevalence of active epilepsy in our study was similar to some other studies from the region. In a prevalence study of active epilepsy in 6–12-year-old children in rural Kenya, the unadjusted prevalence of active epilepsy (defined as at least one convulsion in the previous year) was 3.4 per 1,000 (95% CI 2.4–4.8) (Mung’ala-Odera et al., 2008). In a later study in the same population group, the unadjusted prevalence of active convulsive epilepsy in 6–12-year-old children was 3.0 per 1,000 (95% CI 2.5–3.5) (Edwards et al., 2008). The questionnaire used to detect epilepsy (Placencia et al., 1992) was designed in a different population but did perform nearly as well in the community as in the clinic situation with a high sensitivity and specificity. Assuming a similar sensitivity in our population, the adjusted prevalence would be 3.67 per 1,000.

There have been three other studies in Tanzania that have found a higher prevalence ranging from 8.6 to 10.2 per 1,000 (Rwiza et al., 1992; Dent et al., 2005; Winkler et al., 2009). The differences in prevalence may be due to variation in study populations, study designs, definitions, and exposure to environmental factors. Our study included those who had seizures of all types and we used capture-recapture using a screening questionnaire and key informant methodology that is known to increase the proportion of cases identified within a community and reduce selection bias (Debrock et al., 2000). Therefore, we would have expected a higher prevalence. Of the positive census responders that were not found, three had epilepsy (known to be on antiepileptic drugs). Other cases may not have been brought forward due to stigmatization of those with epilepsy, which is known to occur in Tanzania (Rwiza et al., 1993). In addition, others would be missed if their symptoms were not recognized as epilepsy, especially cases of nonconvulsive epilepsy. Unfortunately we were unable to perform external validation of prevalence because local hospital records were inadequate to accurately identify cases of epilepsy. The unadjusted prevalence in our study was similar to that in developed countries, which is similar to large community-based studies of prevalence in the region (Edwards et al., 2008) and may reflect increased mortality in those with epilepsy and with coexistent disability (Carter et al., 2005).

### Sources of bias

To minimize ascertainment bias, our study aimed to identify all cases of childhood epilepsy in the study area by using a validated screening questionnaire and training enumerators to recognize epilepsy. We were not able to validate the questionnaire in our community. Case status was defined by two pediatric neurologists. The proportion followed was high (92.9% in cases and 88.5% in controls) and the proportion of cases (7.1%) and controls (11.5%) who were not seen was similar (p = 0.49). Both cases and controls were assessed using identical methods and questionnaires. Recall bias of adverse perinatal events was minimized by asking an initial generic question about any difficulties in the perinatal period, which was followed in all cases and controls with the same specific questions about length of labor, crying and feeding after birth, color, and any history of admission to hospital. There were no perinatal notes for the majority of children, so formal assessment of recall bias was not possible. The adjustment used in analysis for adverse perinatal events appeared valid as all odds ratios were similar and none of the probability values changed significantly.

In conclusion, epilepsy in children in this part of sub-Saharan Africa is mostly focal in onset, which suggests that most epilepsy in the region has a symptomatic etiology. Genetic factors may be important, but a history of adverse perinatal events was strongly associated with epilepsy; better antenatal and perinatal care could reduce the burden of epilepsy in the region.
